# Improving Diabetes Care for Young People With Type 1 Diabetes Through Visual Learning on Mobile Phones: Mixed-Methods Study

**DOI:** 10.2196/jmir.2155

**Published:** 2012-08-06

**Authors:** Dag Helge Frøisland, Eirik Årsand, Finn Skårderud

**Affiliations:** ^1^Research Centre for Child and Youth Competence DevelopmentLillehammer University CollegeLillehammerNorway; ^2^Faculty of MedicineUniversity of OsloOsloNorway; ^3^Norwegian Centre for Integrated Care and Telemedicine (NST)University Hospital of North NorwayTromsøNorway; ^4^Department of Computer ScienceUniversity of TromsøTromsøNorway; ^5^Department of Special Needs EducationUniversity of OsloOsloNorway

**Keywords:** Diabetes, adolescents, user-centered design, education, eHealth, mHealth, mobile phones, short message service, SMS, qualitative research, triangulation of methods

## Abstract

**Background:**

Only 17% of Norwegian children and adolescents with diabetes achieve international treatment goals measured by glycated hemoglobin (HbA_1c_). Classic patient–physician consultations seem to be poorly adapted to young children. New strategies that are better attuned to young people to improve support of adolescents’ self-management of diabetes need to be tested and evaluated.

**Objective:**

(1) To explore how applications for mobile phones can be used in follow-up of adolescents with type 1 diabetes, and (2) to use the findings to guide further development of the applications and as a basis for future studies.

**Method:**

We pilot tested two mobile phone applications: (1) an application that contained a picture-based diabetes diary to record physical activity and photos taken with the phone camera of food eaten, where the phone also communicated with the glucometer by Bluetooth technology to capture blood glucose values, and (2) a Web-based, password-secured and encrypted short message service (SMS), based on access using login passwords received via SMS to be used by participants to send messages to their providers when they faced obstacles in everyday life, and to send educational messages to the participants. At the end of the 3-month pilot study, 12 participants (7 girls and 5 boys ) aged 13–19 years completed semistructured interviews. The participants had a mean HbA_1c _value of 8.3 (SD 0.3), mean age of 16.2 (SD 1.7) years, mean body mass index of 23.3 (SD 3.2) kg/m^2^, and mean diabetes duration of 7.5 (SD 4.6) years. We applied three additional measurements: change in metabolic control as measured by HbA_1c_, the System Usability Scale, and diabetes knowledge.

**Results:**

From the interviews, three main categories emerged: visualization, access, and software changes. Participants appreciated the picture-based diary more than the SMS solution. Visualization of cornerstones in diabetes self-care (ie, diet, insulin dosage, physical activity, and pre- and postprandial glucose measurements all transformed into one picture) in the mobile diary was found to be an important educational tool through reflections in action. This led to a change in participants’ applied knowledge about the management of their disease. Additional measurements supplemented and supported the qualitative findings. However, changes in HbA_1c _and participants’ theoretical knowledge as tested by a 27-item questionnaire, based on a national health informatics’ diabetes quiz, before and after the intervention were not statistically significant (*P *= .38 and *P *= .82, respectively, paired-samples *t *test). Participants suggested additional functionality, and we will implement this in the design of the next software generation.

**Conclusion:**

Participants reported an increased understanding of applied knowledge, which seem to positively affect diabetes self-care. Visual impressions seem well adapted to the maturation of the adolescent brain, facilitating the link between theoretical knowledge and executive functions. SMS gave the adolescents a feeling of increased access and security. Participants gave valuable input for further development of these applications.

## Introduction

### Diabetes

Type 1 diabetes is one of the most common chronic illnesses of childhood. The incidence of diabetes in Norway is one of the highest in the world, reported as 32 new cases per 100,000 person-years [1]. Type 1 diabetes often requires rigorous daily routines and a high level of self-management, which is seen as a complex task. Treatment goals might be achieved through frequent *blood glucose* *measurements *as well as through *insulin medication *tailored to *food intake *and *physical activity*. These elements are often described as the cornerstones of diabetes treatment. A significant increase in blood glucose measurement was found in a study using reminders and cueing, social media communication, and gamification [2]. However, in a review of telemedicine, Farmer et al concluded that intervention “with or without telemedicine is only likely to be helpful when test results are linked to educational or behavioral advice and changes in clinical management” [3]. Clinical guidelines highlight the importance of education; nevertheless, this seems to be a missing functionality in most telemedicine interventions [4].

### Adolescence and Diabetes

Adolescence as a transitional phase in human life challenges both young people and their caregivers [5]. Physicians and caregivers as well as adolescents with diabetes often experience frustration and powerlessness when the adolescents repeatedly return to their clinics with poor metabolic control.

Only 17% of Norwegian children and adolescents with diabetes achieve international treatment goals measured by glycated hemoglobin (HbA_1c_) [6]. As researchers, we need to uncover the reasons behind such treatment results and explore ways to improve them. One approach is to develop developmentally appropriate means of supporting adolescents’ self-management of diabetes. Consequently, we developed two mobile phone applications as tools for diabetes self-management. The research described in this paper is an evaluation of these tools.

### Education

The development of competence (ie, knowledge and skills) among people living with diabetes is integral to their effective management of the disease. In modern health care, providers are advised to use new technology and educational models based on learning theory, health models, and instructional design theory when delivering patient education [7]. Diabetes self-care is highly dependent on coordination of thoughts and behavior—that is, executive functions. Skills necessary for such coordination are selective attention, decision making, voluntary response, inhibition, and working memory [8]. Motor and sensory brain areas mature first; the primary visual cortex matures early, while areas involved in executive functions mature later. These development patterns might facilitate the application of modern technology using visual imaging in this particular patient group [9]. Visual impressions have been shown to improve understanding and self-management in patients with chronic diseases [10,11].

### Information and Communication Technology

Information and communication technology (ICT) has developed rapidly over the last decades. Reports from Statistics Norway show that among adolescents aged 13-19 years, more than 90% use the Internet for more than 2 hours every day; 95% have their own mobile phone, and more than half of them accessed the Web through their mobile phone daily [12]. ICT is gradually being applied in health care and eases the flow of information between providers and their patients [13-15]. These technologies include Internet, email, and mobile phone applications, and are often referred to as electronic health or eHealth [16].

The use of ICT to facilitate health care has traditionally been dominated by PC-based technology [14]. Many ICT studies show overall positive results and indicate that ICT-based interventions improve health care utilization, health behaviors, attitudes, skills, and knowledge [14]. Studies involving mobile phones used by adults have been published [15,17-20], but there are few reports of the use of mobile phone-based tools among children and adolescents with type 1 diabetes.

When a new ICT system is developed, user-involved design of patient-operated systems is advocated to promote useful applications [21]. Based on this philosophy, the Norwegian Centre for Integrated Care and Telemedicine (NST) has developed several mobile applications based on user-participatory design processes.

### Aim

The aim of this study was to evaluate adolescent patients’ experiences with two different mobile phone applications used for diabetes care. We wanted to examine whether an intervention using information and communication tools could affect disease management measured by metabolic control and through qualitative methods. We also wanted the adolescents to use their experiences to guide the product developers by giving advice on further improvements. Finally, we wanted to propose new hypotheses for further research on approaches that diabetes teams might apply to facilitate the use of young people’s competencies.

## Methods

### Design

This study used triangulation of methods to provide details about the phenomenon studied that would not be available with the use of one method alone. Interviews were recorded. Field notes were taken systematically during the semistructured interviews and used as an additional data source. Data on metabolic control measured by HbA_1c _were collected before and at the end of the intervention. HbA_1c _was analyzed at the local hospitals using Bayer DCA 2000 (Tarrytown, NY, USA; normal reference range 3.4%-6.1%). Usability data were collected using the System Usability Scale (SUS), a questionnaire related to human-computer interaction consisting of a simple 10-item scale based on a 5-point Likert scale [22]. A SUS score above 58 is regarded as above average, and a SUS score above 80 is regarded as high and a score where participants are likely to recommend the product to friends [23]. Additional data were collected before and at the end of the intervention period through a 27-item questionnaire based on the Norwegian National Health Informatics’ diabetes quiz [24].

### Intervention

The intervention period was 3 months. This included an instruction day when the participants were introduced to an HTC Touch 2 mobile phone (HTC Corporation, Bellevue, WA, USA), on loan from NST, and the two novel diabetes software applications: a mobile phone-based diabetes diary called Diamob and the Diabetes Message System, a short message service (SMS) based on the Secure Health Dialogue system [25]. Participants could use the Diamob application as much as they wanted, but 2 periods of recording data, each lasting 3 days, before the consultation were mandatory. Participants came for a consultation with the research team (midway) to discuss use of the applications and to participate in a reflection-in-action talk about the mandatory 3-day recordings in the Diamob application. When the participants browsed through the pictures, self-reflection was guided by emphasis on recording images of food, level of activity, insulin dosages, and pre- and postprandial glucose measurements. Two physicians from the outpatient clinics were responsible for the Diabetes Message System responses. At the end of the 3-month period, the participants met with the research team for a semistructured interview.

### Technological Applications

#### Application 1

**Figure 1:**
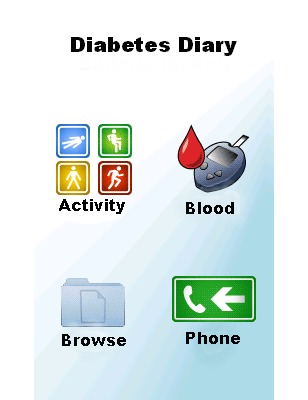
The first step in the Diamob application, consisting of pictograms specifying planned physical activity. This leads the user to the next step.

The prototype Few Touch application [17] was optimized by NST to include a camera-based dietary capture and a feedback component. This modified version of the Few Touch application is referred to as the Diamob application and targets communication between the patient and the health care team about carbohydrate evaluation and insulin dosages. The application was used by adolescents with type 1 diabetes to document the food they ate. They had to choose one of four pictograms describing the physical activity in which they planned to participate or had already been engaged in (Figure 1).

**Figure 2:**
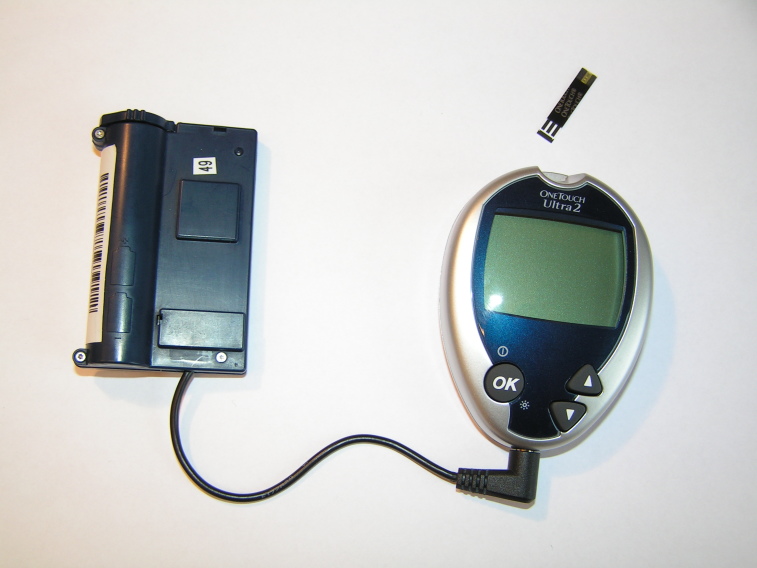
The Bluetooth transmitter (left) sends glucose measurements to the phone as soon as the test strip is extracted from the glucometer.

The page for the first step guided the participants to a screen prompting them for the insulin dosage suitable for the food they planned to eat. The next step was to photograph the food using the mobile phone’s camera. The phone communicated with the glucometer by Bluetooth technology to capture blood glucose values (Figure 2). One and a half hours after the meal, the phone reminded the users to measure their postprandial blood glucose.

**Figure 3:**
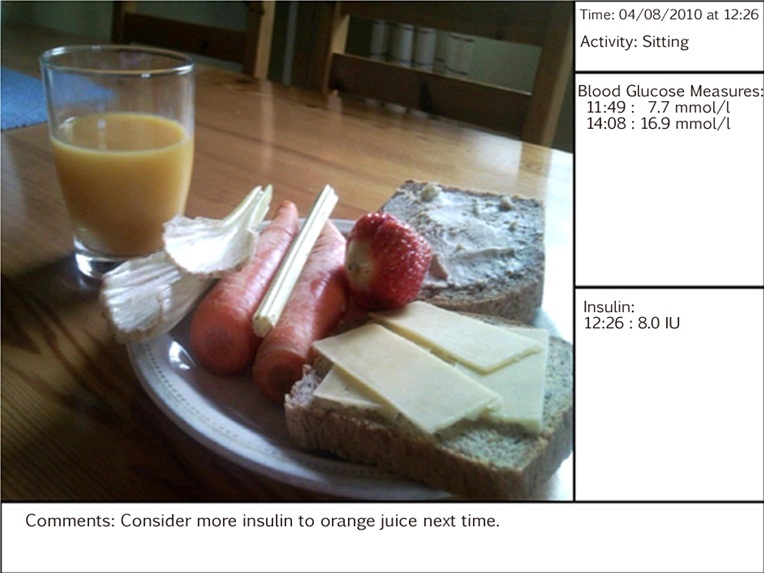
Example of a picture produced by the Diamob application. Pictures were available for users to browse through on the phone and could also be uploaded to computers and used during consultations.

The picture produced by the Diamob application incorporated relevant pre- and postprandial blood glucose values, insulin dosage given, and information about the participant’s physical activity (Figure 3).

#### Application 2

Due to strict Norwegian laws regarding security and privacy of health communication, we created a functional Web-based encrypted SMS based on the Secure Health Dialogue SMS with the help of a commercial software company (WTW AS, Tiller, Norway) [25]. This Web-based SMS system is referred to as the Diabetes Message System. It is based on access using login passwords received via SMS to ensure that health information is not compromised. We invited the participants to use this application to send messages to their providers when they faced obstacles in everyday life. The Diabetes Message System application was also tailored to send educational messages to the participants. An example of such messages is “Reasons for high blood sugar: Not enough insulin, intake of food rich in carbohydrates, infections, fever.”

### Sample

We recruited a convenience sample of adolescents aged between 13 and 19 years from two pediatric clinics in Eastern Norway, Innlandet Hospital Trust. Inclusion criteria were a diagnosis of type 1 diabetes at least 1 year prior to the start of the study and current HbA_1c _of less than 10.0%. We set the upper HbA_1c _limit to avoid bias from potential psychological maladjustment associated with poor metabolic control. We enlisted 12 participants: 7 girls and 5 boys. Of the 12 interviewees, 1 withdrew from the use of the application halfway through the intervention for personal reasons. We interviewed all 12 participants at the end of the study period.

### Interviews

We developed a semistructured interview guide to elicit responses on the topics described in the aims of the study. The guide included questions to identify different experiences with the implemented technology. The interviews took place in a meeting room at the outpatient clinics. They lasted between 45 and 90 minutes. Recordings were later transcribed, including notes on nonverbal aspects of the communication, such as pauses and laughter.

### Analytical Procedures

The qualitative data analysis was based on transcribed interviews, supported by the interview moderator’s field notes. The analytic approach was based on qualitative description [26], influenced by phenomenology and hermeneutics [27]. The analysis entailed an inductive process, focusing on the participants’ experience with the mobile device in an attempt to make sense of their personal experiences with the device. The understanding of another person’s situated experience of the world requires interpretation, thus making hermeneutics an important part of the approach.

According to a bottom-up principle, all the interviews were carefully read and reread with the aim of discriminating units of meaning from the transcripts. Such units were assigned codes. Elements of texts with the same codes were identified and extracted to code sheets. Units of meaning were first identified, and condensed units of meaning were then developed into categories. Finally, the codes and categories were organized hierarchically.

To increase validity and reduce possibilities of idiosyncratic coding, two of the authors conducted the analysis separately [28]. The categories were discussed and negotiated until consensus was achieved. In the final stage of the process, these concepts were tested by comparing them with the transcripts, according to a top-down principle, to ensure validation of the extraction. Through this process, the final categories emerged. To ensure that the ensuing concepts were well represented in the data, the research team discussed the developing analysis to add credibility to the study and ensure agreement on the main themes.

### Statistical Analyses

Results are presented as means with 1 standard deviation (SD). Paired-samples *t *test analyses were used to compare pre- versus postinterventional data. Significance was defined as *P *< .05. We used SPSS version 18.0 (IBM Corporation, Somers, NY, USA) for analyses.

### Ethical Considerations

The adolescents and their parents gave written consent for the study according to Norwegian requirements. The study was approved by the regional committee for medical research ethics (ref. 2009/773b). As compensation, participants could ask for a refund of the costs of their Internet subscription to cover expenses related to accessing the Web-based SMS application

## Results

A total of 7 girls and 5 boys participated. Their mean HbA_1c _was 8.3 (SD 0.3) before the intervention. Mean age was 16.2 (SD 1.7) years, mean disease duration was 7.5 (SD 4.6) years, and mean body mass index was 23.3 (SD 3.2) kg/m^2^. All the participants used insulin pumps, compared with 75% in the background population. All participants used a mobile phone daily, and 8 (67%) reported accessing the Internet via a mobile phone daily. The participants were asked to commit to completing 2 sets of diabetes diary records, each covering a continuous period of 3 days, during the intervention. This would yield approximately 24 pictures for each participant (288 pictures). During the study, 691 pictures were downloaded (mean 50, minimum 25, maximum 94).

The overall result from the qualitative data demonstrated that the adolescents found both mobile applications useful as a support to their diabetes self-management. They appreciated the picture based diabetes application more than the SMS solution. Table 1 give a brief overview of the process of analyzing the interviews, from which three main categories emerged: visualization, access, and software changes. The quotes used to illustrate these themes use fictitious names for the participants.

**Table 1:** Brief overview of the process of analyzing the interviews.

Theme	Codes and condensed meaning	Category and concept final theme	Findings and hypothesis
Functionality of mobile phone diary Communication facility	To see the coherence of treatment Communication through pictures To visually identify unhealthy food	Visualization	Diamob seems to be more an educational tool to enhance understanding of diabetes self-care than just a communication tool. This might be due to brain development among children and adolescents, and because practical executive competence is facilitated by visual memory rather than theoretical facts.
SMS^a^ Educational messages	Closeness Empowerment Easy access to information	Access	SMS is a useful and preferred tool for adolescents to enhance contact with the health care system, and a nonencrypted version is favored.
Technical advice and challenges	Technical challenges Future development	Software changes	Changes to the two applications need to be made to increase functionality.

^a ^Short message service.

### Visualization

All the adolescent participants used the verb “to see” in relation to the first application’s functionality. They reported a better visual understanding of the cornerstones of diabetes self-management: food intake, insulin dosage, physical activity, and blood glucose measurements. The participants reported that the pictures of food they had consumed, integrating pre- and postprandial glucose measurements and insulin dosages as well as information on physical activity, gave them a visual and tangible understanding of how physical activity, food intake, and the insulin dosage interact and affect postprandial glucose measurements.

Tom Before, I really thought that the blood sugar was one thing and giving insulin was one thing and eating was one thing, but now I see more all three of them as a whole, that they all belong together. Because if not all three of them come together, I feel it’s like I miss a part of the puzzle.

Ann It is just to browse back in the picture diary and look at how much [insulin] I actually needed to the food I had eaten, that is an advantage...I have learned to think about what to eat and the value of measuring my blood sugar before I eat and think about the insulin dosage in relation to these factors, then to measure again 1.5 hours later in order to evaluate if the dosage of insulin was correct.

Erik The Diamob application helped the most...I became tougher in taking insulin doses. Because I saw how the glucose measurements changed and the value of giving enough insulin.

The participants also said that they preferred the mobile phone-based diabetes diary to paper-based diaries, and that it provided an incentive for communication about their diabetes self-management both with their parents and with their health care providers.

Oda I think it is a lot easier to understand and to have it explained when I can see things.

Beth When I met Mom or Dad I could show them what I had done during the day instead of writing. I liked that a lot.

The adolescents also reported that seeing the pictures of their own food in the Diamob application gave them a visual understanding of their own unhealthy diet.

Erik I just photographed the food I usually eat, but I thought during the process that I should apply more healthy eating habits, because I saw I had a lot of unhealthy canteen food in school.

### Access

The participants experienced Web-based SMS as a positive instrument for bidirectional contact with health care providers. It provided a safety net that gave them a sense of protection because it made it easy for them to access their physician with questions and concerns.

Jan I liked the project and the follow-up. I could send an SMS whenever I wanted. I got an answer within half an hour. I especially liked the SMS—in the Netherlands, where I lived prior to this, I knew I could call, but I like the message system a lot better.

Oda The fact that you have someone to support you—someone who knows the subject, and if you get into difficulties you can get an answer—it gives a certain feeling of security.

All participants greatly appreciated the possibility of contacting their health care provider and receiving an immediate response. They indicated that it gave them a feeling of closeness to the health care practitioner.

Tom It is usually not easy to get in touch with your doctor...it was nice, because usually you call in and you are told he is for lunch or are busy and then it ends up you don’t do anything.

The participants also reported feeling empowered in that they could access the health care practitioner so readily.

Beth It has been pretty good to know that if I have an issue, then I can just send a message...Instead of calling Mom or Dad and ask them to call [the physician], and when they have the answer it might be an answer to something I was not wondering about.

The participants reported that they appreciated having access to information through the mobile phone. They described the educational text messages as useful in increasing their understanding of diabetes self-management. However, they also stated that they appreciated simple and practical self-management advice more than large amounts of information that was not relevant to their immediate situation.

Oda It is more about those messages and the information. It has been practical advice, easy to understand, simple facts that are very nice to know. It is better to have it in such small portions instead of reading a lot of information, then everything is poorly read and poorly understood. I liked the way the information was given.

Kristin ...it is easier to access the information in this way, than listening to a doctor who talks about it for 5 hours.

Despite their appreciation for the accessibility of the device, several participants indicated that the Web-based SMS system was unduly cumbersome.

Jan But it was a bit cumbersome to have to remember a code [to get access].

David But what is cumbersome is that you have to access that Internet browser on the mobile. I would prefer to send normal SMS on the phone...that would make it even easier if you could access it using the usual SMS [on the phone].

### Software Changes

Another category of data was the software changes that the participants recommended. Although the participants described the applications as highly usable in general, they also identified particular problems that required resolution. They reported technical problems that we attributed to the immaturity of the applications and the chosen mobile platform. Some of the participants explained that they experienced problems and found it troublesome to use the applications.

Jan The Diamob app didn’t work at the end of the project. The glucometer with Bluetooth worked, but batteries ran out of power quickly.

David Yes, the project was tailored to me, but it could have been better on the glucose transmission [from the glucometer to the phone] because it didn’t work all the time.

The participants agreed that the applications were highly usable, but requested the possibility of downloading the applications to their own mobile phones in the future. With 1 exception, the participants indicated that if the applications were available for downloading to their own phones, they would continue to use the applications. They contributed technical advice on how the applications could be improved.

Oda It would give a better overview as a matter of fact, when you think about it, when you have to go from picture to picture, but if you had all 3 days in 1 page, as a complete overview of 1 day, then I think it would be better.

Eva I didn’t like the diagram thing [in the Diamob apps]. It was a mess, and I didn’t like that it could not be tailored to each patient...And for instance I think it is stupid that it [the pictograms for activity] only marks sitting, lying, standing, and training, but maybe you don’t do the physical activity, and then, after an hour, it is time to go for a run.

### Additional Measurements

Qualitative data were supported by glycemic control, SUS scores, and knowledge tests. Metabolic control had improved (ie, lowered HbA_1c_) in 7 participants at the end of the study, but was unchanged in 2 participants. During the study period, 3 of the participants increased their HbA_1c _(Table 2). Statistically, we found no significant changes in HbA_1c _over the course of the intervention (Table 3). None of the participants who completed the 3-month intervention had severe hypoglycemia or severe ketoacidosis or were hospitalized during the intervention period.

The mean score on the SUS was 73 (SD 22) (Table 2); 10 participants had a high SUS score: mean 81 (SD 10). These 10 reported positive experiences with the 2 mobile applications. However, 2 participants had a considerably lower SUS score than the others (each scoring 30).

All the participants had relatively high scores on the theoretical diabetes knowledge tests, with a mean of 22 (SD 2) (maximum score 27; Table 2). Scores were nearly the same prior to the project and at the end; no statistical differences were found (Table 3).

**Table 2:** Participants’ glycated hemoglobin (HbA_1c_) and knowledge test score (maximum score 27), before and after the intervention, and System Usability Scale (SUS) score (maximum score 100) after the intervention.

Participant	HbA_1c _%		Knowledge test score		SUS score
	Before	After	Before	After	
Jan	8.6	9.0	18	20	67.5
David	6.6	6.4	22	21	87.5
Eva	8.6	7.6	11	NA^a^	30
Beth	8.3	8.3	23	23	87.5
Kristin	8.0	8.4	21	22	65
Oda	7.4	7.2	23	23	87.5
Tom	9.4	9.1	21	24	30
Erik	9.2	8.3	23	24	92.5
Ann	8.6	8.6	23	27	80
Emma	7.1	7.0	25	21	95
Ingrid	8.9	8.5	18	18	75
Trond^b^	9.0	9.7	24	20	77.5

^a ^Eva did not take the postintervention knowledge test, and this is marked as not available.

^b ^Trond did not complete the whole intervention for personal reasons unrelated to the study and was interviewed at the end.

**Table 3:** Glycated hemoglobin (HbA_1c_) values, knowledge test scores, and System Usability Scale score before and after the intervention.

Measure		Mean	SD	No.	*P *value (paired- samples *t *test)
**HbA** **1c**					.38
	Before	8.3	0.9	12	
	After	8.1	0.9	12	
**Knowledge test score**					.82
	Before	22.0	2.3	12	
	After	22.1	2.5	11	
**System Usability Scale score**					
	All participants	73.0	22.1	12	
	High scorers^a^	81.5	10.2	10	

^a ^Mean score excluding the 2 participants who scored low on the System Usability Scale (score of 30).

## Discussion

The most surprising finding was the reports of a new visual understanding of diabetes treatment. Visual impression and consultations based on reflection in action seemed to have a startling effect on self-reported perception of the coherence of the cornerstones of diabetes treatment: diet, insulin dosage, physical activity, and blood glucose measurements. Most of the adolescents considered the mobile picture diary to be superior to paper-based systems and preferred the SMS application as a convenient means of communicating with their health care providers.

### Limitations

A limitation of the study is that our sample might be biased. Those who volunteered to participate might have been more comfortable with the technology than those who did not. The intervention period was short, and some technical problems arose. Although the sample is small, statistical analyses were performed on some of the variables. Low numbers of participants and the short intervention period reduce the potential for proving statistical significance. A control group would have strengthened this potential. The technical solutions had limitations in regard to both applications and mobile phones. The Diamob application was only compatible with the HTC 2 mobile phone, and participants disliked that phone’s touch-sensitive user interface. Furthermore, they experienced problems with Bluetooth transfer of the glucose measurements.

### Visualization

The last 20 years of diabetes education have reflected an increased emphasis on integrated educational strategies and collaboration with the patient [29,30]. Future mHealth applications should implement the educational benefits of visualization with the report of factors that enhance the frequency of glucose measurements [2].

Despite several large multicenter randomized studies on diabetes treatment, we have limited knowledge about why adherence to diabetes treatment, especially among young people, is so difficult. Applications like the Diamob application may help to bridge the gap between theoretical knowledge and lack of execution of treatment competencies in different contexts in patients’ everyday life. The use of meal images and the reflection-in-action method contributes to the change in applied knowledge and ease of communication during consultation [31]. Health care personnel need to recognize patients’ existing competencies, experiences, and preferences to be able to deliver education and health services tailored to the people they serve. Simple visual tools designed by young people in their own personal settings seem important to improve patients’ comprehension, recall, and adherence [10,11], which our study also supports. In their review article of visual impression in patient education, Houts et al [10] asserted that pictures with cultural relevance that were designed by patients themselves are suitable for increasing understanding and adherence to treatment [10]. The Diamob application creates such personal pictures, and the widespread use of mobile phones among adolescents in Norway should encourage the use of such applications in this population [12].

To perform well in self-care, patients depend on well-developed executive functions: the ability to store important information, to keep in mind a plan to carry out in the future, and to inhibit impulses. Work has associated these behaviors with the frontal lobes, with maturation late in the adolescent period [8]. Magnetic resonance imaging studies have shown that maturation of the visual cortex and the subsequent pruning of neural connections happen early in childhood, compared with the frontal parts of the brain, where centers for cognitive functions such as planning and advanced thinking are located [8,9].

It has been shown that children with type 1 diabetes have slightly lower cognitive function than healthy children, and that they perform less well than controls on global measures of both intelligence and neuropsychological skills such as attention and executive functions [32]. The lack of maturation and pruning in the adolescent brains might explain the mismatch between theoretical competence and execution of practical skills found in our study. Professionals educating young people with diabetes need to be aware that they are less able than adults to link theoretical knowledge with cognitive and executive functions.

### Access

Young people rarely approach health care providers by themselves, and often communicate to physicians and other practitioners through their parents. It has been claimed that questions considered difficult to ask in a face-to-face dialogue, or that should not be postponed until the next clinical appointment, are facilitated by text messaging [33]. As in the Sweet Talk study [34], the participants in our study reported using the SMS solution relatively rarely; nevertheless, they appreciated its function as a safety net and a feeling of increased access to their health providers. Before the study, the participating professionals expressed a fear that SMS would be overused, but these assumptions proved unfounded. Our Web-based, password-secured, encrypted message system prevented leaks of health information through mobile phone communication. Such systems might be appreciated all over the world to ensure that health information is not compromised.

Global penetration of mobile phones is increasing rapidly, with a penetration rate of up to 90% in high-income countries. This suggests the potential for using mobile phone applications in the treatment of young people. Future research into patients’ own experiences of this feeling of access and its significance for treatment results, quality of life, and a sense of empowerment is recommended.

### Technical Considerations

Technical problems were reported by the participants in the study and need to be taken into consideration for future versions of this application. Novel hardware and software already on the market based on other mobile platforms may ease the transfer challenges, overriding the need for Bluetooth technology. We appreciated the suggestions for additional functionality, for instance, new presentation software, and will try to implement this when designing the next software generation. A whole body of literature supports participatory design as mutually beneficial for both end users and developers [35-37].

### Additional Findings

It seems that with a few reservations, the findings from the qualitative interviews were supported by the additional methods used to evaluate this study. The triangulation of methods, with the use of semistructured interviews, SUS, and knowledge questionnaires in relation to metabolic control (HbA_1c_), strengthened this study. This provides additional information and allows for greater accuracy. An additional strength of the study is that it gave a voice to the experiences and concerns of the adolescents themselves. Previous work has shown that our health care system is poorly designed to meet the needs of patients with chronic diseases, and that Web-based programs and ICT tools are useful in meeting these patients’ requirements [38,39]. Our findings contribute to this literature [20,40].

### Conclusion

The integration of cornerstones in diabetes treatment into a picture-based diary gave young people a better understanding of their diabetes treatment. Furthermore, it is more an educational tool than a communication tool. We hypothesize that the reported effect of the picture-based diary is partly due to early maturation of the visual cortex of the brain. Members of diabetes care teams need to take into account that the child and adolescent brain is immature, a work in progress. It is likely that young people are less capable than we previously thought of converting different theoretical facts related to diabetes into applied knowledge in their daily lives.

Participants reported the Web-based encrypted SMS application for mobile phones as a sought-after tool that gave them a feeling of access and security. Participants gave valuable input for further development of these applications.

Simple information and communication tools like the applications in this study should be further developed and tested in larger-scale studies to investigate their role as a mediator for increased understanding and better self-care for diabetes in the adolescent population.

## Abbreviations

HbA1c: glycated hemoglobin
ICT: information and communication technology
NST: Norwegian Centre for Integrated Care and Telemedicine
SMS: short message service
SUS: System Usability Scale
